# Supplementary material for: Characterization of Parthanatos in Breast Cancer: Implications for Prognosis and PARP Inhibitor Resistance
Source: Bioengineering (Basel). 2025 May 29;12(6):586. doi: 10.3390/bioengineering12060586 (PMC12189614; doi:10.3390/bioengineering12060586)
Supplement: Supplementary file 1 [file bioengineering-12-00586-s001.zip › bioengineering-3644598-supplementary.pdf]

Supplementary Material

Article

Characterization of Parthanatos in Breast Cancer: Implications for Prognosis and PARP Inhibitor Resistance

Junjie Tang1,2*, Qian Liu1*, Wei Du3*, Linxi Chen4, Feiyang Qi4, Ranxin Zhang5, Bang H. Hoang5, David S. Geller5, Rui Yang5, Jichuan Wang4#, and Li Hu1#

[1] Key laboratory of Carcinogenesis and Translational Research (Ministry of Education), Familial & Hereditary Cancer Center, Peking University Cancer Hospital & Institute, Beijing 100142, China.
[2] The First Clinical Medical School, Nanjing Medical University, Nanjing, China.
[3] Breast Center, Peking University People's Hospital, Beijing 100044, China
[4] Musculoskleletal Tumor Center, Beijing Key Laboratory for Musculoskeletal Tumors, Peking University People's Hospital, Beijing 100041, China
[5] Department of Orthopedic Surgery, Montefiore Medical Center, Albert Einstein College of Medicine, Bronx, NY.*

Correspondence: Jichuan Wang, MD, Ph.D., Musculoskleletal Tumor Center, Beijing Key Laboratory for Musculoskeletal Tumors, Peking University People's Hospital, Beijing 100041, China, E-mail:jcwang@pku.edu.cn

Li Hu, MD, Ph.D., Familial & Hereditary Cancer Center, Peking University Cancer Hospital& Institute, Beijing, 100142, P. R. China, E-mail: 2165610821@bjmu.edu.cn

*These authors have contributed equally to this work and share first authorship.

Table S1. Comparison of clinical characteristics between C1 and C2 subtypes.

	Total	C1	C2	P-value
**No. of patients**	**1082**	**503**	**579**	
**Age at diagnosis**				0.024
Median (range)	58 (26-90)	60 (28-90)	57 (23-95)	
Mean ± SD	58.4 ± 13.2	59.4 ± 13.1	57.5 ± 13.2	
**Ethics**				0.225
White	678 (74.8%)	325 (77.2%)	353 (72.6%)	
African American	171 (18.9%)	74 (17.6%)	97 (20.0%)	
Asian	58 (6.4%)	22 (5.2%)	36 (7.4%)	
NA	175	82	93	
**Gender**				0.133
Female	1070 (98.9%)	500 (99.4%)	570 (98.4%)	
Male	12 (1.1%)	3 (0.6%)	9 (1.6%)	
**Histology**				0.015
IDC	678 (74.6%)	334 (77.0%)	334 (71.4%)	
ILC	198 (21.8%)	93 (21.4%)	105 (22.4%)	
Medullary	5 (0.6%)	3 (0.7%)	5 (1.1%)	
Metaplastic	8 (0.9%)	1 (0.2%)	7 (1.5%)	
Mucinous	17 (1.9%)	2 (0.5%)	15 (3.2%)	
Mix	3 (0.3%)	1 (0.2%)	2 (0.4%)	
NA	173	69	104	
**Molecular subtype**				0.210
ER/PR+HER2-	598 (63.5%)	284(64.7%)	314 (62.4%)	
HER2+	163 (17.3%)	66 (15.0%)	97 (19.3%)	
TNBC	181 (19.2%)	89 (20.3%)	92 (18.3%)	
NA	140	64	76	
**Tumor Size**				0.652
T1	216 (26.6%)	97 (24.6%)	119 (28.5%)	
T2	457 (56.4%)	229 (58.1%)	228 (54.7%)	
T3	105 (12.9%)	52 (13.2%)	53 (12.7%)	
T4	33 (4.1%)	16 (4.1%)	17 (4.1%)	
NA	271	109	162	
**Lymph nodes metastases**				0.257
N0	429 (48.0%)	213(49.5%)	216 (46.6%)	
N1	301 (33.7%)	135 (31.4%)	166 (35.8%)	
N2	103 (11.5%)	56 (13.0%)	47 (10.1%)	
N3	61 (6.8%)	26 (6.0%)	35 (7.5%)	
NA	188	73	115	
**Distant metastases**				0.453
M0	1001 (98.2%)	467 (97.9%)	534 (98.5%)	
M1	18 (1.8%)	10 (2.1%)	8 (1.5%)	
NA	63	26	37	
**TNM stage**				0.837
Stage 0	0 (0.0%)	0 (0.0%)	0 (0.0%)	
Stage I	176 (17.3%)	79 (16.6%)	97 (17.9%)	
Stage II	610 (59.9%)	286 (60.0%)	324 (59.8%)	
Stage III	215 (21.1%)	102 (21.4%)	113 (20.8%)	
Stage IV	18 (1.8%)	10 (2.1%)	8 (1.5%)	
NA	63	26	37	
**MKI67 mRNA level**				<0.001
Median (range)	1700 (16-11824)	1915 (16-11648)	1546 (92-11824)	
Mean ± SD	2288 ± 1955	2491 ± 2014	2111 ± 1887	

Abbreviations: SD, standard deviation; IDC, invasive ductal carcinoma; ILC, invasive lobular carcinoma; NA, not available or not applicable; ER, estrogen receptor; PR, progesterone receptor; HER2, human epidermal growth factor receptor 2; TNBC, triple-negative breast cancer. 、

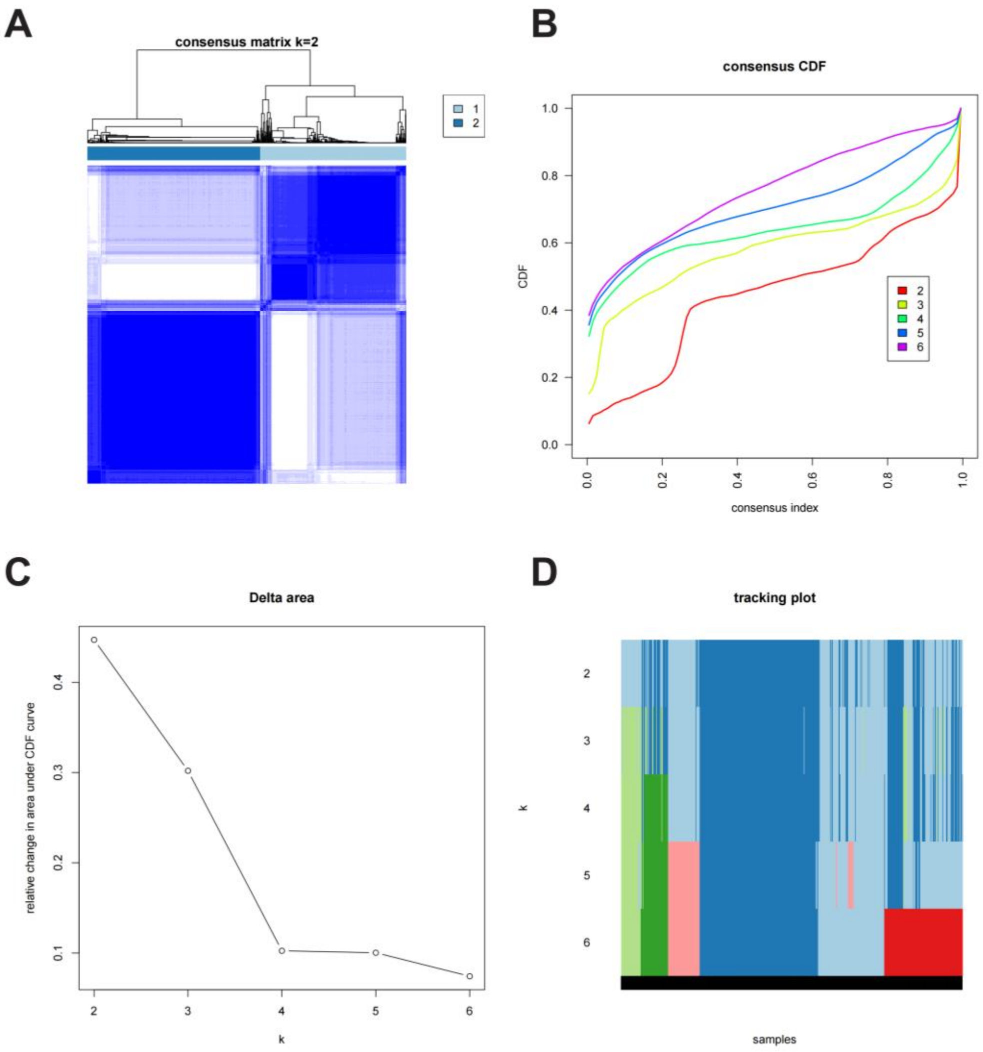

Figure S1. Details of clustering analysis. (A) A consensus matrix heat map of k = 2 clusters and their associated regions. (B) Cumulative distribution function (CDF) curve under different cluster numbers. (C) The relative change in area under the CDF curve for different values of k. (D) Tracking plot of samples in different values of k.

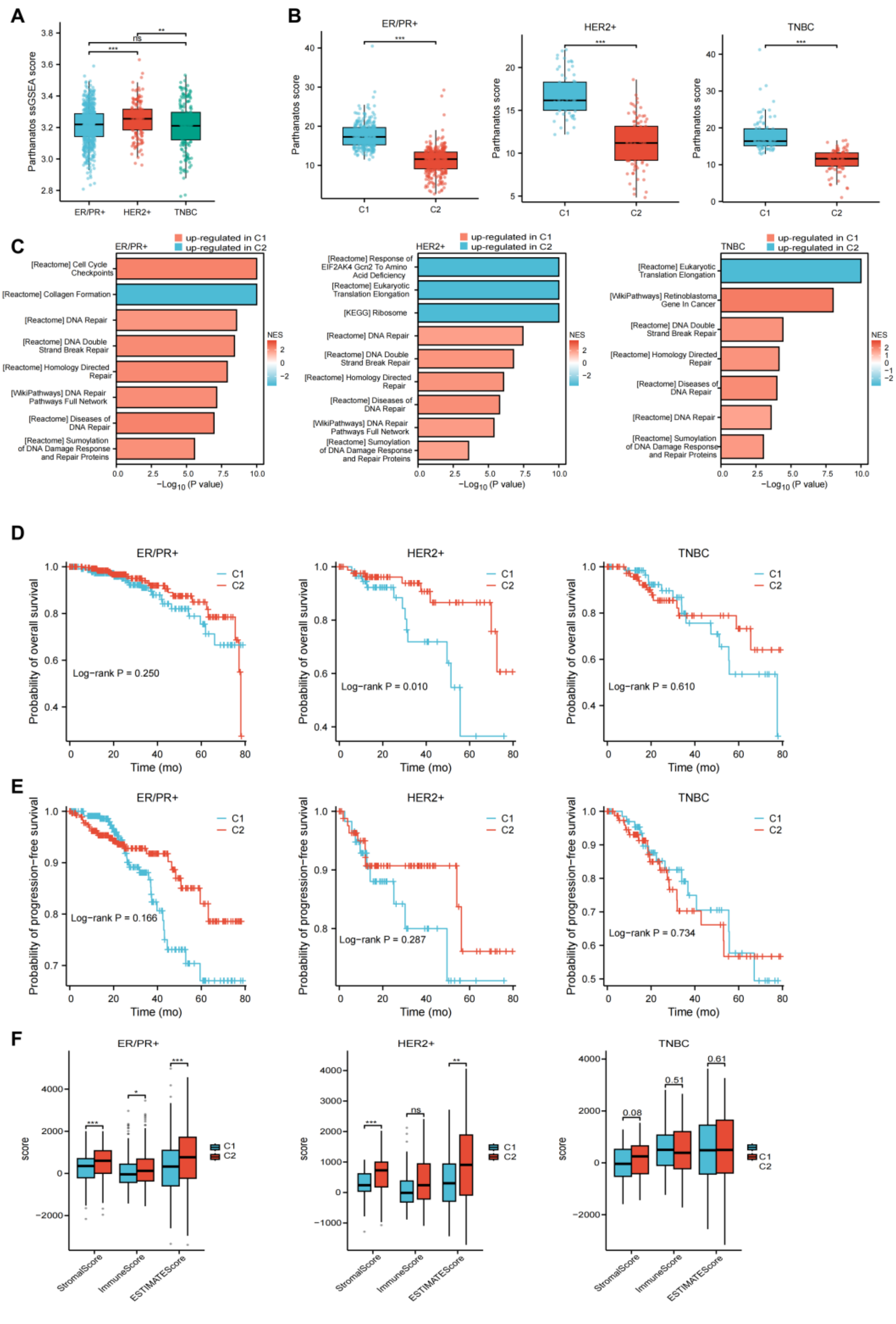

Figure S2: Parthanatos-based subtypes across different breast cancer subtypes. (A) Box plots showing parthanatos pathway scores across different breast cancer subtypes, with p-values calculated using the Wilcoxon rank-sum test. (B) Box plots showing parthanatos pathway scores for two clusters (C1 and C2) across different breast cancer subtypes, with p-values calculated using the Wilcoxon rank-sum test. (C) Gene set enrichment analysis between C1 and C2 subtypes across different breast

cancer subtypes. (D–E) Kaplan–Meier survival analyses of C1 and C2 subtypes across different breast cancer subtypes. (F) Differences between the two clusters in the ESTIMATE algorithm across different breast cancer subtypes, with p-values calculated using the Wilcoxon rank-sum test. Abbreviations: Mo, months.

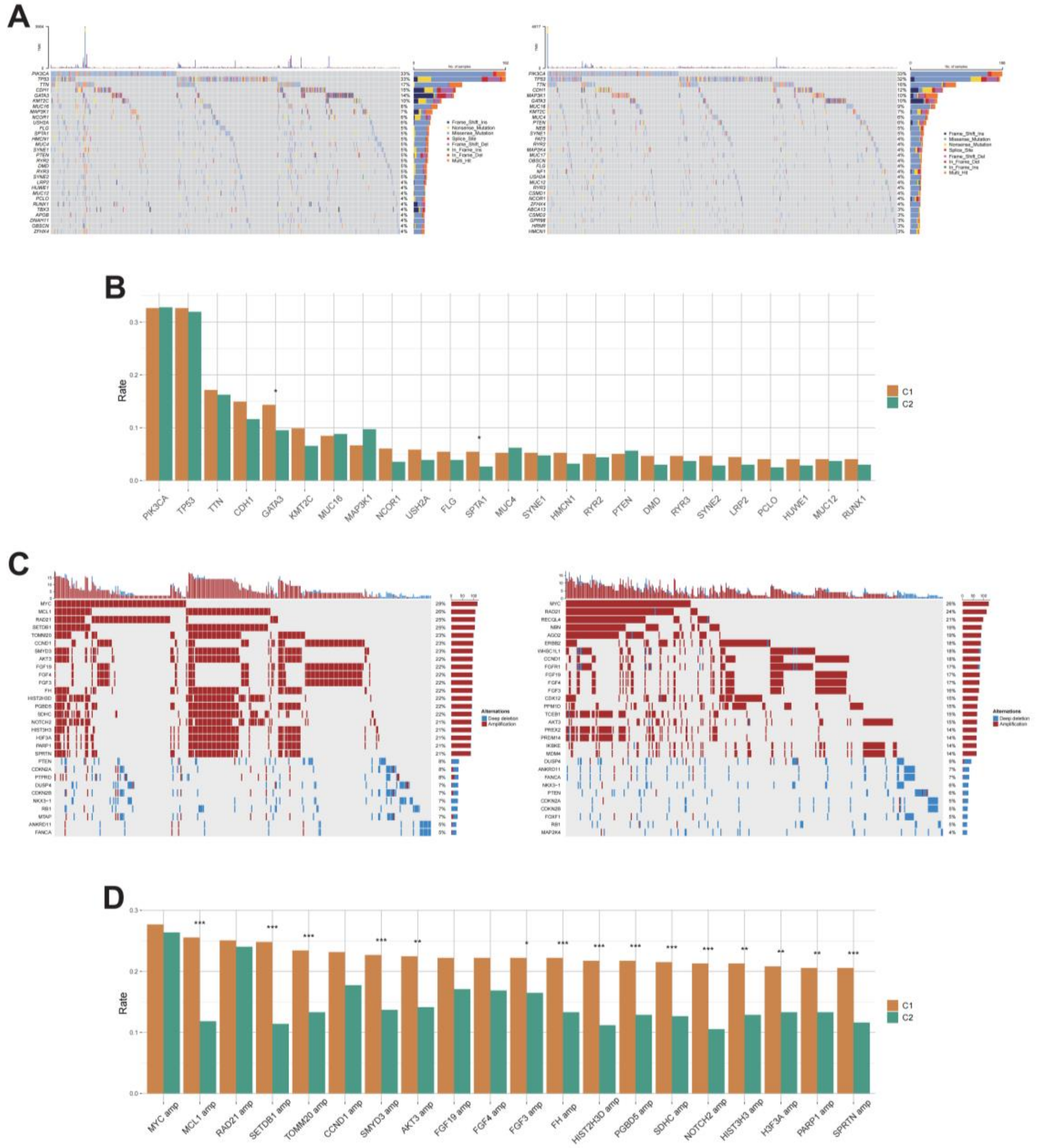

Figure S3: Somatic mutation profiles in parthanatos-based BC subtypes. (A) Mutation profiles of C1 (left) and C2 (right) subtypes. Genes are ordered by mutation rate; TMB (mut/Mb) is recorded on the top of the plot. (B) Comparison of gene mutation rates between C1 and C2 subtypes. (C) Profiles of copy number changes in C1 (left) and C2 (right) subtypes. (D) Genes exhibiting a significantly increased rate of copy number changes in the C1 subtype compared to the C2 subtype. Abbreviations: AMP, amplication.

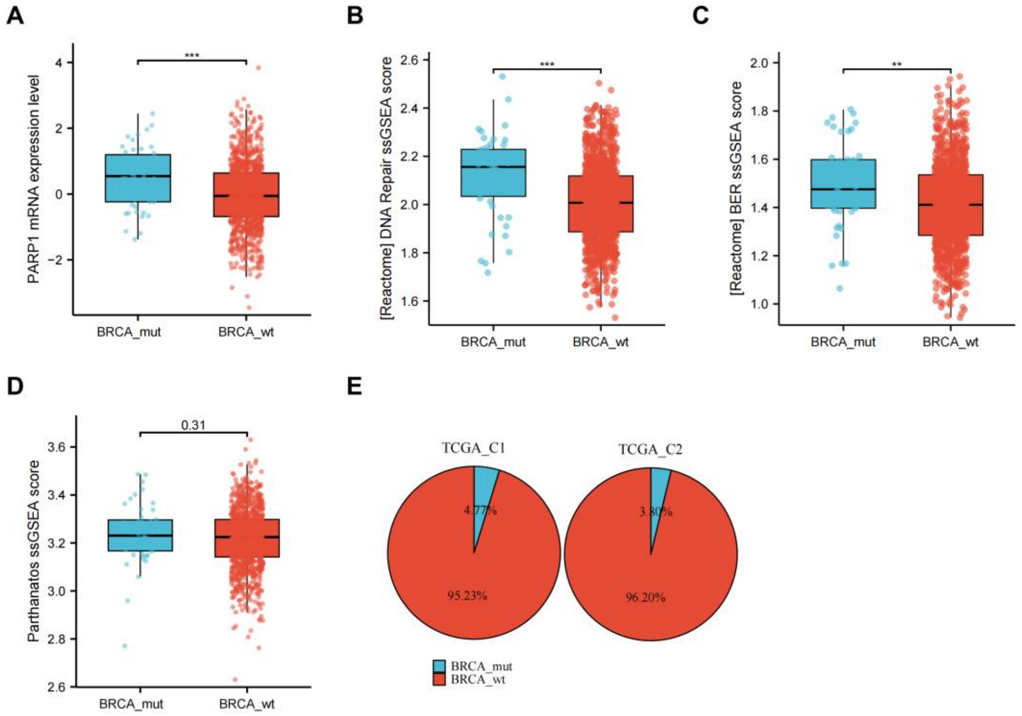

Figure S4: Association between parthanatos-based subtypes and *BRCA* mutations in breast cancer. (A) mRNA expression levels of the PARP1 gene in *BRCA*-mutant breast cancers and *BRCA*-wildtype cases from TCGA databases, with p-values calculated using the Wilcoxon rank-sum test. (B) Box plots showing DNA repair pathway scores between *BRCA*-mutant breast cancers and *BRCA*-wildtype cases, with p-values calculated using the Wilcoxon rank-sum test. (C) Box plots showing base excision repair pathway scores between *BRCA*-mutant breast cancers and *BRCA*-wildtype cases, with p-values calculated using the Wilcoxon rank-sum test. (D) Box plots showing parthanatos pathway scores between *BRCA*-mutant breast cancers and *BRCA*-wildtype cases, with p-values calculated using the Wilcoxon rank-sum test. (E) The proportion of *BRCA*-mutant cases between C1 and C2 subtypes from TCGA databases. Abbreviations: MUT, mutant; WT, wildtype; BER, base excision repair.
